# Diet Selection Exhibited by Juvenile and Adult Lifestages of the Omnivores Western Tarnished Plant Bug, *Lygus hesperus* and Tarnished Plant Bug, *Lygus lineolaris*

**DOI:** 10.1673/031.010.12701

**Published:** 2010-08-06

**Authors:** James R. Hagler, C. Glen Jackson, Jacquelyn L. Blackmer

**Affiliations:** United States Department of Agriculture, Agricultural Research Service, Arid-Land Agricultural Research Center, Maricopa, Arizona, USA

**Keywords:** *Bemisia tabaci*, cotton, feeding behavior, predation, herbivory

## Abstract

*Lygus hesperus* Knight and *Lygus lineolaris* (Palisot de Beauvois) (Heteroptera: Miridae) are economically important plant bugs on many crops worldwide. However, these omnivores are also facultative predators on a wide variety of insects. This study was conducted to quantify and compare herbivory and carnivory exhibited among different lifestages of these two insect pests. The feeding activity of a total of 422 individuals was observed for 1 h each in feeding arenas containing a cotton leaf disk and copious amounts of the sweetpotato whitefly, *Bemisia tabaci* (Gennadius) (Hemiptera: Aleyrodidae) eggs, nymphs and adults. The *L. hesperus* and *L. lineolaris* lifestages examined included adults and 3^rd^, 4^th^ and 5^th^ instar nymphs. Plant feeding occupied the majority of both species' time budget, regardless of the species or lifestage examined. There was a tendency for *L. lineolaris* lifestages to feed more often and for longer duration on plant tissue than *L. hesperus.* All lifestages of both species rarely fed on *B. tabaci,* but when they did, they preferred nymphs > adults > eggs. There were only a few cases where there were significant differences in predation rates and prey handling times exhibited among lifestages and between species, but juvenile *L. hesperus* tended to be more predaceous than juvenile *L. lineolaris* on whitefly nymphs and adults and 5^th^instar and adult *L. lineolaris* were significantly more herbaceous than their *L. hesperus* counterparts. In addition, the younger individuals of both species tended to have greater prey handling times than their older counterparts. The significance of these findings is discussed.

## Introduction

The western tarnished plant bug, *Lygus hesperus* Knight and the tarnished plant bug, *L. lineolaris* (Palisot de Beauvois) (Heteroptera: Miridae) are serious pests of a wide variety of crops including cotton, alfalfa, beans, safflower, stone and pome fruit, and strawberries (Jackson et al. 1995). While these mirids are notorious pests, they also are aggressive predators (Wheeler 1976). Predation events by various *Lygus* species have been reported on a wide variety of prey including various Lepidoptera, Hemiptera, and beneficial species (Champlain and Sholdt 1967; Lindquist and Sorenesen 1970; Wheeler 1976, 2000; Cleveland 1987; Hagler and Naranjo 1994; Pfannenstiel and Yeargan 2002; Hagler et al. 2004; and many others). A comparative study of the digestive enzyme complexes of adult *L. hesperus* and *L. lineolaris* females showed that both species have enzyme complexes that are adapted for omnivory (Agustí and Cohen 2000). However, this study suggested that although both species are more adapted for herbivory than carnivory, *L. hesperus* “might be more suited as a predator than *L. lineolaris.*” Moreover, it was determined that survival of *L. hesperus* on insect-free alfalfa was poor and improved when aphids were present (Butler 1968) and that it was much easier to rear *L. hesperus* when beet armyworms were added to their plant diet (Bryan et al. 1976). To date, the most successful artificial diets developed for *Lygus* contain both plant-derived and animal-derived nutrients in the mixture (Debolt 1982; Cohen 2000). Clearly, there is an abundance of literature documenting the omnivorous feeding activity of *Lygus* bugs (see Wheeler 2001 for a review).

The goal of this study was to quantify and compare the feeding activity of *L. hesperus* and *L. lineolaris* to corroborate the digestive enzyme physiology work of Agustí and Cohen (2000). Specifically, we quantified the degree of herbivory and carnivory exhibited between the immature (3^rd^ through 5^th^ instar) and adult lifestages of *L. hesperus* and *L. lineolaris* in feeding arenas that contained a cotton, *Gossypium hirsutum* (L.), leaf disk hosting sweetpotato whitefly, *Bemisia tabaci* (Gennadius) (a.k.a. silverleaf whitefly, *B. argentifolii* Bellows and Perring) (Hemiptera: Aleyrodidae) eggs, nymphs, and adults.

## Materials and Methods

### Lygus bug rearing and maintenance

The *L. hesperus* used in this study originated from alfalfa, *Medicago sativa* L. and cotton fields located at the University of Arizona-Maricopa Agricultural Center, Pinal County, Arizona, USA. The *L. lineolaris* used in this study originated from wild host plants; *Erigeron annuus* (L.) Per soon, *Oenothera speciosa* Nuttall, *Rumex crispus* L., and *Conyza canadensis* (L.) and cotton in Washington, Sunflower, Leflore, and Bolivar counties, Mississippi, USA. Both *Lygus* species were first reared for successive generations (> 10) under standard environmental conditions (14:10 L:D at 27° C, 30% RH) on an artificial diet containing both plant and animal derived nutrients (Debolt 1982). Oviposition packets yielded from each main *Lygus* colony were then placed into separate rearing containers that contained sprouted potatoes, *Solanum tuberosum* L. (Slaymaker and Tugwell 1982) or artificial diet (Debolt 1982) and held under the same environmental conditions described above. Those *L. hesperus* and *L. lineolaris* treatments reared on potato sprouts were reared for ≥ two consecutive generations on the potato diet prior to the behavioral observations. Neonate *L. hesperus* and *L. lineolaris* were allowed to feed freely on each diet until they reached the targeted lifestage for testing.

### Feeding arena

The behavior of *L. hesperus* and *L. lineolaris* individuals ranging in stage from third instar to adult were monitored continuously for 1 h per individual in a feeding arena containing a cotton leaf disk infested with whitefly eggs, nymphs (various instars), and adults as described by Hagler et al. (2009). Cotton plants (cv ‘*Delta Pine 5415*’) were grown in 15.2-cm diameter pots in a greenhouse using standard cultural practices. Four- to 5-week-old plants were infested with adult whiteflies on a weekly basis. When the plants were ≈8 weeks old, a single cotton leaf containing numerous whitefly eggs and nymphs was removed from a plant and cut to fit exactly into the bottom of a 3.5-cm plastic Petri dish (the feeding arena). The number of whitefly eggs and nymphs on the leaf were counted and the leaf was placed abaxial side up into the bottom of the feeding arena. Then, ≈40 adult whiteflies were added to the arena. A typical arena contained a 3.5-cm cotton leaf disk infested with an average (± SD) of 465.9 ± 476.1 whitefly eggs, 402.2 ± 353.4 whitefly nymphs of various stages, and 39.7 ± 11.9 whitefly adults (1:1 sex ratio).

### Behavioral observations

All *Lygus* bugs were removed from their rearing containers within 24 h after they reached the desired lifestage for testing and placed individually in 9.0-cm Petri dishes containing a wet sponge. Individuals were held overnight (e.g., 12 to 16 h without food) prior to observation. On the day of observation, an individual *Lygus* bug (3^rd^, 4^th^ or 5^th^ instar or adult) was placed into the feeding arena and continuously observed for 1 h under a dissecting microscope. After each 1 h observation, the *Lygus* bug was removed from the arena and replaced with another *Lygus* bug. One hour observations were conducted intermittently throughout the day from 0600 to 1500 h in a room with controlled temperature (25° C) and humidity (25%). Due to the large sample sizes and nature (e.g., waiting for the *Lygus* to reach the desired lifestage for testing) of the experiment, the observations were recorded over a long time span from 7 April through 3 November, 1999. Two to 6 individuals of each species were tested each day for a total of 214 *L. hesperus* individuals and 208 *L. lineolaris* individuals. No more than two *Lygus* were observed consecutively in the same arena. The feeding arenas were replaced daily with fresh plant and prey material. Preliminary observations revealed several distinct events in the feeding arenas; these behaviors were programmed into The Observer® (Noldus, www.noldus.com). Descriptions of the *Lygus* behaviors that were recorded are given in Table 1. The proportion of time spent walking, resting, grooming, and orienting were very similar for each lifestage and species, therefore these behavioral events were combined into an “other” behavioral category to simplify the data presentation. The distinction between a *Lygus* bug feeding and probing event was determined by the length of time that the mouthparts were inserted into the prey or plant tissue. Specifically, if the mouthparts were inserted into a food item for < 5 sec, it was designated as a probing event (e.g., exploratory) and if the mouthparts were inserted for ≥ 5 sec, it was designated as a feeding event (e.g., committed to feeding). This timeframe was arbitrarily chosen based on the time that we felt that a *Lygus* bug committed to a feeding event (e.g., most of the probing events were < 3 sec in duration while the vast majority of feeding events were much greater that 5 sec in duration).

### Data analysis

To evaluate feeding habits within and between *L. hesperus* and *L. lineolaris,* differences in diet choice (whitefly lifestage or plant) and feeding duration were tested among *Lygus* nymphs and adults. It was hypothesized that the different diets that the *Lygus* were reared on (e.g., artificial diet vs potato diet) would affect *Lygus* bug diet choice in the feeding arenas. However, in initial statistical analysis the diet rearing history did not have a significant effect on the feeding behavior of either *Lygus* species' therefore data from the potato and artificial diet rearing treatments were pooled by species and lifestage for all further statistical analyses. Similarity, there were no significant differences in the feeding activity among adult males and females of each species; therefore these data were pooled for each species.

Differences in *Lygus* feeding activity on each whitefly lifestage and on the cotton plant between each *L. hesperus* and *L. lineolaris* lifestage were first analyzed for statistical differences by a Student's *t*-test (SigmaStat, Ver. 3.5, www.sigmaplot.com). When the data did not fulfill the assumptions of a normal distribution or equal variance as determined by the SigmaStat software, the non-parametric Mann-Whitney Rank Sum Test was used to identify significant differences in feeding activity between each lifestage. Similarly, differences in the frequency and duration of feeding events within the lifestages (3^rd^, 4^th^, 5^th^ instar and adult) of each *Lygus* species did not meet the assumptions of ANOVA. Therefore, a Kruskal-Wallis one-way ANOVA on ranked data was used to identify significant differences in feeding frequency and feeding duration among the lifestages of each species. When a significant difference was detected, means were separated using the Dunn's multiple comparison test (SigmaStat, Ver. 3.5).

**Table 1.**
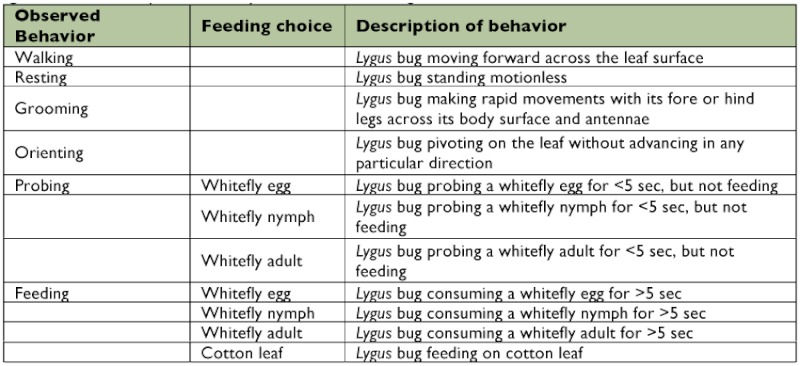
Description of the behavioral events recorded for Lygus *hesperus* and L *lineolaris* exposed to a cotton leaf disk containing the various sweetpotato whitefly, *Bemisa tabaci* life *stages.*

## Results

**Figure 1.**
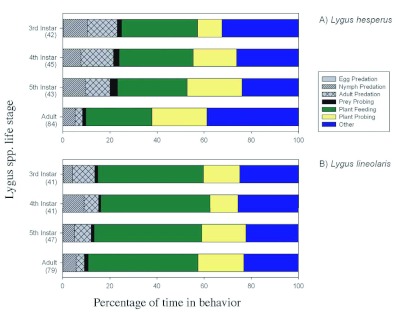
Behavioral time budgets exhibited for 3^rd^ instar through adult Lygus *hesperus* and *L. lineolaris* in a feeding arena containing a cotton leaf disk and whitefly eggs, nymphs and adults. Results are expressed as the percentage of total time spent in each behavioral element. The “other” behavioral category is the pooled proportional amount of time that each *Lygus* species and lifestage spent walking, resting, grooming and orienting. The number in parentheses on the y-axes are the sample sizes. High quality figures are available online.

The proportional amount of time that *L. hesperus* and L. *lineolaris* spent in the various behavioral categories is summarized in Figure 1. Plant feeding and plant probing events occupied ≈50% of the time for each of the *L. hesperus* lifestages examined. Immature *L. hesperus* lifestages spent about 22% of their time engaged in predation, compared to *L. hesperus* adults, which engaged in predation ≈10% of the time. The remaining 25 to 30% of *L. hesperus* time was spent in the other behavioral categories. The time spent in the other behavioral categories was similar between lifestages with ≈8.3, 11.0, 11.0 and < 1.0% of their time spent grooming, resting, walking and orienting, respectively (Figure 1A). Herbivory also occupied the majority of the *L. lineolaris* lifestages time budget with 65% of their time dedicated to plant feeding and plant probing events. The immature *L. lineolaris* lifestages spent about 14% of their time feeding on whiteflies, which was about 1.5 times longer than that of the adults (Figure 1B). About 25% of the remaining *L. lineolaris* time budget was spent in the other behavioral categories. Moreover, as with *L. hesperus,* the time spent in each behavior category was similar between the lifestages. Specifically, *L. lineolaris* spent ≈7.0, 10.0, 7.0 and < 1.0% of their time grooming, resting, walking and orienting, respectively (Figure 1B).

**Figure 2.**
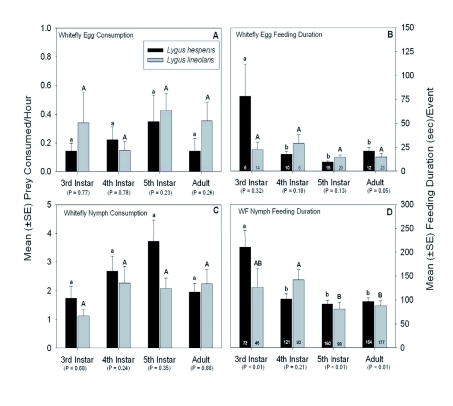
(A) Mean (±SE) number of whitefly eggs consumed by Lygus *hesperus* and *L. lineolaris.* (B) Mean (±SE) amount of time it took an individual to consume a single whitefly egg. (C) Mean (±SE) number of whitefly nymphs consumed by *L. hesperus* and *L. lineolaris.* (D) Mean (±SE) amount of time it took for an individual to consume a single whitefly nymph. (E) Mean (±SE) number of whitely adults consumed by *L. hesperus* and *L. lineolaris.* (F) Mean (±SE) amount of time it took for an individual to consume a single whitefly adult. (G) Mean (±SE) number of plant feeding events exhibited by *L. hesperus* and *L. lineolaris.* (H) Mean (±SE) amount of time for an individual plant feeding event. Different lower case letters above the error bars indicate significant differences in feeding activity among the four *L. hesperus* lifestages and different upper case letters above the error bars indicate significant differences in feeding activity among the four *L. lineolaris* lifestages as determined by the Kruskal-Wallis ANOVA on ranks followed by the Dunn's multiple comparison test (test statistics are provided in the text). The *P*-value for each paired comparison denotes differences in feeding activity between each *L. hesperus* and *L. lineolaris* lifestage as determined by either the Student's *t*-test (when the assumptions of the *t*-test were fulfilled) or, in most instances, the Mann-Whitney rank sum test. The sample sizes for the feeding consumption data (all plots on the left side of the graph) are presented in Figure 1. The numbers inside the vertical bars (on all the plots on the right side of the graph) are the total number of observations recorded over the duration of the study. High quality figures are available online.

The average numbers and durations of *Lygus* herbivorous and predaceous feeding events recorded during the one hour observations are given in Figure 2. *Lygus* bugs, regardless of the lifestage or species examined, rarely fed on whitefly eggs. Overall, < 0.5 eggs were consumed per hour of observation (Figure 2A). There were no significant differences in the average number of eggs consumed between each lifestage of the two species or among the lifestages of each species (Kruskal-Wallis ANOVA of ranks test, H = 4.87, df = 3, P = 0.182 for *L. hesperus* and H = 7.18, df = 3, P = 0.066 for *L. lineolaris,* respectively; the *P* values for each pairwise comparison is given in Figure 2A). With the exception of third instar *L. hesperus,* all individuals preying on whitefly eggs usually spent < 25 sec to consume an egg. Specifically, 3^rd^ instar *L. hesperus* took significantly longer to feed on an egg than did the older counterparts (H = 8.0, df = 3, P = 0.047), but there were no significant differences in the amount of time that it took other *L. lineolaris* lifestages to consume a whitefly egg (H = 4.8, df = 3, P = 0.19). The only significant difference in feeding duration on whitefly eggs between the two *Lygus* species was exhibited by the adult lifestage. Adult *L. hesperus* took significantly longer to consume an egg than adult *L. lineolaris* (Figure 2B), but these data should be interpreted with caution due to the small number of feeding events recorded over the duration of the study.

**continue:**
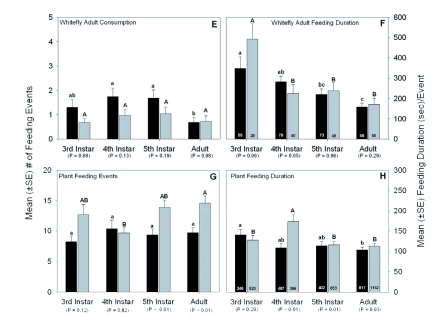

Both *Lygus* species were most often observed preying on whitefly nymphs than the other stages. A total of 932 nymphal whiteflies (518 for *L. hesperus* and 414 for *L. lineolaris*) were consumed over the course of 422 hours of direct observation. There were no significant differences in the frequency of predation events on whitefly nymphs among the lifestages of each *Lygus* species (H = 5.7, df =3, P = 0.126 for *L. hesperus* and H = 2.6, df = 3, P = 0.457 for *L. lineolaris,* respectively) or between the any lifestage of the two species (Figure 2C). The average time for both *Lygus* species to consume a nymph ranged from 100 and 150 sec for most lifestages (Figure 2D).

Third instar *L. hesperus* took significantly longer to consume an individual whitefly nymph than it did its older counterparts (H = 10.679, df = 3, P = 0.014); and 4^th^ instar *L. lineolaris* took longer to feed on nymphs than its 5^th^ instar and adult counterparts (H = 10.082, df = 3, P = 0.013). These data also showed that the feeding durations between species were significantly longer for 3^rd^ and 5^th^ instar and adult *L. hesperus* than for its *L. lineolaris* counterparts (Figure 2D).

Predation events by *Lygus* on the adult whitefly lifestage were relatively rare over the course of the study with ∼1 individual consumed per hour of observation (Figure 2E). A total of 440 adult whiteflies (265 for *L. hesperus* and 175 for *L. lineolaris)* were consumed during the study. There were no significant differences in the number of adult whiteflies consumed among the various *L. lineolaris* lifestages (H = 3.94, df = 3, P = 0.268). However, 4th and 5th instar *L. hesperus* preyed on significantly more adult whiteflies than did adult *L. hesperus* (H = 14.782, df = 3, P = 0.002; Figure 2E). There were no significant differences in the number of adult whiteflies consumed between lifestages of the two *Lygus* species. The feeding duration for *Lygus* on an adult whitefly generally lasted from 200 and 300 seconds (Figure 2F), regardless of the lifestage or species examined. Third instar *L. hesperus* and *L. lineolaris* fed longer on an adult whitefly than did their older counterparts (H = 8.828, df = 3, P = 0.032 for *L. hesperus* and H = 15.730, df = 3, P = 0.001 for *L. lineolaris;* respectively). The only significant difference in feeding duration between the two species was exhibited by the 4^th^ instar lifestages. Specifically, 4th instar *L. lineolaris* fed at a significantly faster rate than 4^th^ instar *L. hesperus* (Figure 2F).

As mentioned above, herbivory was the dominant behavior exhibited by *L. hesperus* and *L. lineolaris.* A total of 4,753 plant feeding events were recorded during the study. Over all lifestages combined, there was an average of 13.1 ± 0.7 (n = 2,721) and 9.5 ± 0.5 (n = 2,032) plant feeding events recorded per hour of observation for *L. lineolaris* and *L. hesperus,* respectively. There were no significant differences in the number of plant feeding events among the various *L. hesperus* lifestages (H = 1.048, df = 3, P = 0.790), but 4^th^ instar *L. lineolaris* fed significantly less often on plant tissue than the adult lifestage (H = 9.804, df = 3, P = 0.020) (Figure 2G). Moreover, 5th instar and adult *L. lineolaris* fed significantly more often on the cotton plant than their *L. hesperus* counterparts. The average plant feeding duration for both *Lygus* species lasted > 100 seconds (Figure 2H). The plant feeding duration for 3^rd^ instar *L. hesperus* was significantly longer than for adults (H = 14.695, df = 3, P = 0.002) and for 4th instar *L. lineolaris* than 3^rd^ instar, 5^th^ instar and adult *L. lineolaris* (H = 43.774, df =3, P < 0.001). The feeding durations between the lifestages of each species revealed that 4^th^ instar, 5^th^ instar, and adult *L. lineolaris* fed significantly longer on cotton leaf tissue than its *L. hesperus* counterparts (Figure 2H).

## Discussion

The genus *Lygus* is a widely distributed pest of many cropping systems in much of the Northern Hemisphere. *Lygus hesperus* has been reported to feed on almost 150 different host plants worldwide (Schwartz and Foottit 1998); whereas *L. lineolaris* has been reported to feed on over 325 host plants in North America alone (Young 1986). These species are strongly associated with a polyphytophagous life style. However, the fitness of these omnivores can be significantly increased when an insect is added to their diet (Cohen 2000). This is confirmed by several studies that show *Lygus* with an apparent need for animal protein (Butler 1968; Bryan et al. 1976; Wheeler 1976, 2001; but see Rosenheim et al. 2004). Various *Lygus* species have been shown to feed on a wide variety of arthropods including aphids (Lindquist and Sorensen 1970), whiteflies (Hagler and Naranjo 1994), lepidopteran eggs and larvae (Parker 1970; Bryan et al. 1976; Wheeler 1976; Cleveland 1987; Hagler and Naranjo 1994; Pfannenstiel and Yeargan 2002), and various natural enemies (Wheeler, 1976). Cannibalism is also common with *Lygus* (Wheeler 1976; JRH personal observation).

Wheeler (2001) emphasized that more studies are needed to examine the basic food requirements of the mirid taxa. The present study was conducted to quantify and compare the diet choice of *L. hesperus* and *L. lineolaris* in feeding choice arenas containing a cotton leaf disk and the various lifestages of *B. tabaci.* This feeding behavior study was designed to complement previous enzyme physiology research conducted on these two species (Agustí and Cohen 2000). In that study, the salivary and midgut digestive enzyme complex present in the adult female lifestage of both *Lygus* species were compared. Results showed that these two omnivores contain digestive enzymes that are better adapted for phytophagy than zoophagy. However, the authors concluded that *L. hesperus* has digestive enzymes that might make it a better predator than *L. lineolaris* (Agustí and Cohen 2000). Ultimately, our study was designed to quantify the amount of phytophagy and entomophagy exhibited among different lifestages of each species and between lifestages of the two species. Moreover, we wanted to determine if previous rearing history (e.g., preconditioning on a fixed food source) had an effect on *Lygus* diet selection.

Cohorts of both species were reared for several generations on either a strict herbaceous diet consisting only of sprouted potato tubers or on artificial diet consisting of various plant and animal nutrients. It was hypothesized that the different diets would affect *Lygus* diet choice in the feeding arenas. There were not any differences detected in feeding activity and choice of food source based on their previous rearing history. Previous studies also showed that rearing history had little or no effect on the diet selection exhibited by the predator, *Geocoris punctipes.* Those studies showed that *in* vitroreared *G. punctipes* exhibited similar predation patterns on various prey types as their wild counterparts after being reared continuously on a meat-based artificial diet for > 50 generations (Hagler and Cohen 1991) and similar omnivorous feeding patterns as their wild counterparts after being reared in the laboratory on a natural diet (e.g., water, green beans and Lepidoptera eggs) for > 40 successive generations, respectively (Hagler 2009).

The behavioral time budget for each lifestage of both species was clearly dominated by plant feeding and plant probing events. However, there was a subtle difference in the total amount of time that *L. hesperus* and *L. lineolaris* spent feeding on plant and insect tissue that support the findings of Agusti and Cohen (2000). Specifically, *L. hesperus* spent more time feeding on whitefly prey and less time feeding on plant tissue than *L. lineolaris* (Figure 1). The data also showed that the prey feeding duration of both *Lygus* species decreased as the age of the *Lygus* bugs increased. In all likelihood this was a function of the predator to prey size ratio. That is, large predators generally have shorter prey handling times than small predators (Sabelis et al. 1992)

The relatively rare *Lygus* predation events reported are similar in pattern, frequency, and duration as previously reported for adult *L. hesperus* (Hagler et al. 2004). In that study, adult *L. hesperus* were most commonly observed feeding for extended periods of time on a cotton leaf than on any of the whitefly lifestages. Moreover, they consumed only 65, 4, and 3 whitefly nymphs, adults, and eggs, respectively over 27 h of observation. Another study also showed that plant feeding was the predominant behavior exhibited by adult *L. lineolaris* exposed to cotton, alfalfa, and mustard plants (Hatfield et al. 1983). Conversely, another study reported that third instar *L. hesperus* spent more time resting than feeding on a variety of host and non-host plants (Cline and Backus 2002). It should be noted that neither of these two studies included a prey choice in their investigations.

While our study and others (see Wheeler 1976, 2001 for reviews) clearly show that *Lygus* species are highly phytophagous insects, we caution that this laboratory study should not be considered a complete representation of what happens in nature. More comprehensive studies are warranted that compare the diel feeding activity of *Lygus* species exposed to a wider variety of host plant and prey items with variable nutritional rewards. For instance, *Lygus* may be more predaceous during the night than during the day (Pfannenstiel and Yeargen 2002). Also, *Lygus* are known to feed preferentially on plant meristematic tissue (e.g., immature flower buds) over other regions of plants (Scales and Furr 1968; Hanny 1977; Layton 2000). Moreover, adult and late instar *L. hesperus* spend more time feeding on cotton squares than 2^nd^ and 3^rd^ instars (Zink and Rosenheim 2005). The presence of a more desirable host plant(s) (e.g., alfalfa, various weeds, etc.) (Graham et al. 1982, 1986) or feeding site(s) on a host plant (e.g., meristematic tissue) could result in an increased incidence of phytophagy. Conversely, a more nutritious prey type could lead to an increased incidence of carnivory. Omnivory is obviously an important component to the nutritional ecology of mirids and many other heteropterans (Lalonde et al. 1999; Cohen 2000; Agusti and Cohen 2000; Naranjo and Gibson 1996; Wiedenmann et al. 1996; Coll and Guershon 2002). While facultative carnivory is common among many omnivorous true bugs (Polis et al. 1989; Rosenheim et al. 1995; Polis and Winemiller 1996), it is surprising that a greater research effort has not been made towards determining what advantages in fitness are derived by “herbivores” that also feed on arthropods and “predators” that also feed on plants.

Additional research is needed to quantify the feeding behavior, life history, and nutritional requirements of *Lygus* species under varying experimental conditions (Wheeler 2001; Wiedenmann and Wilson 1996). An excellent example of such work has been conducted on the spined soldier bug, *Podisus maculiventris,* where prey scarcity had a negative effect on the development of the omnivore (Wiedenmann and O'Neil 1990; Legaspi and O'Neil 1993, 1994; Crum et al. 1998).

*Lygus* are among the most economically important pests in the world, yet their facultative predation on arthropods also compels further investigation of their nutritive ecology. There are a wide variety of biologically-based pest control alternatives to conventional insecticides that can be better exploited to take advantage of the *Lygus* omnivorous feeding lifestyle (see Stern et al. 1964; Rhainds and English-Loeb 2003; Zink and Rosenheim 2005; Hagler and Blackmer 2007), but a thorough knowledge of their feeding requirements is the foundation for implementing an environmentally benign pest management program.
